# Evolutionary cancer-favoring engineered vaccinia virus for metastatic hepatocellular carcinoma

**DOI:** 10.18632/oncotarget.17288

**Published:** 2017-04-20

**Authors:** So Young Yoo, Su-Nam Jeong, Dae Hwan Kang, Jeong Heo

**Affiliations:** ^1^ BIO-IT Foundry Technology Institute, Pusan National University, Busan 609-735, Republic of Korea; ^2^ Research Institute for Convergence of Biomedical Science and Technology, Pusan National University Yangsan Hospital, Yangsan 626-770, Republic of Korea; ^3^ Department of Internal Medicine, College of Medicine, Pusan National University and Medical Research Institute, Busan 602-739, Republic of Korea

**Keywords:** hepatocellular carcinoma (HCC), oncolytic virus, cancer-favoring vaccinia virus, Wyeth strain, metastasis

## Abstract

Engineered vaccinia virus-based therapy shows promising results in patients with advanced hepatocellular carcinoma, although a strategic virus design for the metastatic liver and the study of its efficacy in treating the cancer has not been well assessed. In this paper, we proposed a simple and strategic virus design for targeting metastatic hepatocellular carcinoma. We developed an evolutionary cancer-favoring engineered vaccinia virus (CVV, which is produced by repeated selective replication in cancerous tissues and then deleting viral thymidine kinase genes) and investigated its therapeutic effects on metastatic liver cancer. The expression of the cell surface marker, CD44, which is associated with cancer stem cells, seems to be correlated with the cells’ metastatic characteristics; cellular migration, epithelial-mesenchymal transition (EMT) expression and liver tumorigenicity. The highly metastatic and tumorigenic Sk-Hep-1 cell line was selected and injected directly onto the liver tissue to develop a liver-to-colon metastasis model. In an animal study, the subjects were treated with sorafenib, CVV, or sorafenib with CVV. Metastatic regions were interestingly rare in the CVV-treated groups (i.e., CVV or sorafenib with CVV) whereas metastatic regions existed in the sorafenib-treated group. From results, we concluded that our simple strategy of developing a cancer-favoring virus can successfully eradicate metastatic liver cancer cells, provided that our CVV can be a promising therapeutic virus that targets metastatic liver cancer.

## INTRODUCTION

Many anticancer drugs have been developed for cancer treatment during the past 30 years; however, most solid tumors remain incurable once they become metastatic. Hepatocellular carcinoma (HCC) is a common solid cancer and the third most frequent cause of cancer-related mortality worldwide. The 5-year relative survival rate for patients with HCC is only 7%, and very few patients survive for more than 1 year [1, 2]. Its correlation with vascular invasion, metastasis, and recurrence leads to poor prognosis of HCC [3]. Metastasis is of great concern and occurs in more than one-half of patients with HCC. Hepatocellular cancer highly metastasizes to distant sites [4]. In addition to the rising incidence of HCC and its poorly understood pathogenesis, HCC is resistant to conventional chemotherapy. There is only one United States Food and Drug Administration (FDA)-approved drug, sorafenib, for systemic use in unresectable HCC [3].

Engineered vaccinia virus-based therapy has recently shown promising results in treating patients with advanced HCC [5–7]; however, its efficacy in treating metastatic liver cancer has not been well assessed. Engineered vaccinia virus (VV)-based therapy has unique merits over conventional anticancer reagents in terms of its tumor selectivity and ability to cause cancer cell lysis. Tumor selectivity is usually introduced by genetic engineering, which attenuates viral replication in normal cells. Strategy relies on disrupting genes that are essential for the virus to replicate in normal cells but that are redundant for replication in cancer cells. The most popular of these has been the disruption in the gene encoding the vaccinia thymidine kinase enzyme (vTK). Deletion of the gene encoding thymidine kinase, an enzyme needed for nucleic acid metabolism, results in dependence of viruses such as VV on cellular thymidine kinase expression, which is high in proliferating cancer cells but not in normal cells. vTK gene-deleted recombinant VVs have demonstrated enhanced tumor selectivity in a number of *in vivo* tumor models [8–10].

In our previous study, clinically applied JX-594 (an engineered vaccinia virus) conferred tumor selectivity via viral thymidine kinase (vTK) inactivation because the vaccinia virus had evolved to replicate in epithelial growth factor receptor (EGFR) pathway-activated cells, which are usually cancer cells with high cellular thymidine kinase levels [9–12]. Thus, engineered VV can selectively replicate in cancer cells. VV comprises the replication competent. Thus, the infectious progeny generated by VV replication in tumor cells spreads to kill the tumor mass, whereas it rarely harms normal cells.

In this study, we investigated an evolutionary cancer-favoring engineered vaccinia virus, CVV [9], which is produced by repeated selective replication in cancerous tissues and then the deletion of viral thymidine kinase genes, as a strategic therapeutics for a metastatic liver cancer. We hypothesized that the higher cancer favoring affinity of CVV can provide much higher cancer selectivity so that it can efficiently find and kill metastasized liver cancers. Four liver cancer cell lines—HepG2, SNU354, SNU449, and Sk-Hep-1—were tested to establish the metastatic liver cancer model.

## RESULTS

### Generation of the CVV

**Figure 1:**
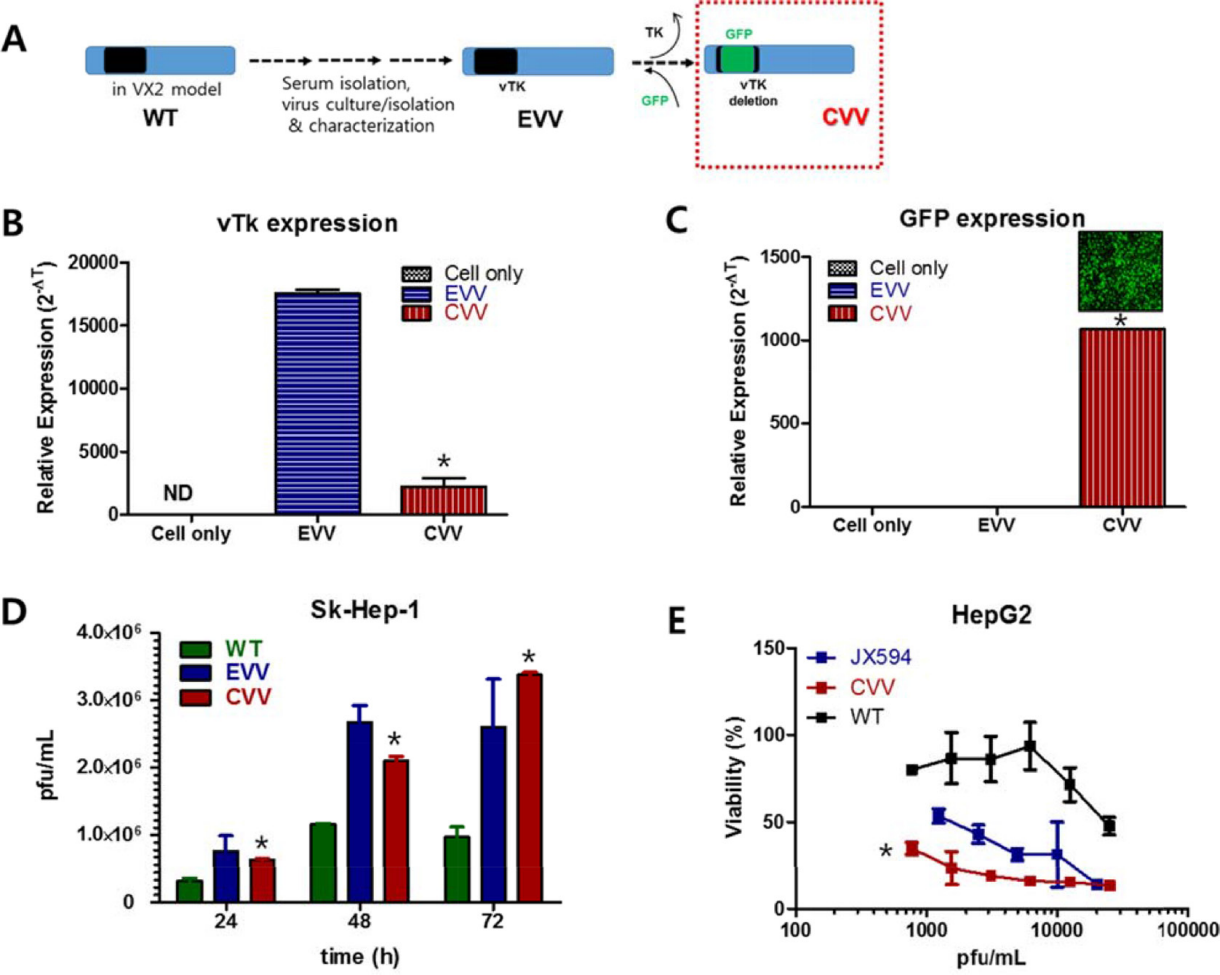
CVV and its higher replication efficiency and cytotoxicity in HCC cell line (**A**) Constructing CVV from EVV and WT. (**B** and **C**) Attenuated vTk expression and GFP expression in CVV-infected cells (*p <0.0001). (**D**) Viral replication assay of WT, EVV and CVV in Sk-Hep-1. CVV deficient of vTk replicated at lower level at 24 h post-infection, but showed higher replication rates than EVV (*p < 0.005). (**E**) Cytotoxicity of CVV, JX594 and WT in HepG2. CVV showed higher cytotoxicity than another VV deficient of vTK such as JX594 (*p < 0.0001). VV, vaccinia virus; CVV, cancer-favoring engineered vaccinia virus; EVV, evolved vaccinia virus; WT, wild type; HCC hepatocellular carcinoma; vTK, viral thymidine kinase.

The CVVs were generated by deleting the vTK gene from naturally evolved cancer-favoring Wyeth strain vaccinia virus (EVV) [14] and inserting *GFP* instead (Figure 1). The EVV was constructed from the Wyeth strain vaccinia virus to achieve its cancer-favoring property. It was then isolated and characterized by repeated replication and tumor tissue lysis [10, 14]. The EVV was isolated from the blood of a vaccinia virus-injected VX2 tumor model when the tumor size reduced and began to release viruses into the serum. In a previous study, we found that the EVV had superior tumor selectivity, compared to the wild type virus and other engineered vaccinia viruses [10, 14]. Attenuated viral thymidine kinase (vTk) expression and ∼99% GFP expression was examined in CVV- infected HeLa cell (Figure 1B and 1C). Replication efficacy generally reflects the antitumor activity and was examined by using Sk-Hep-1 cells. CVV has cancer favoring affinity and vTk deficiency. In terms of cancer favoring, CVV [9] and EVV [14] are comparable because they are evolved from WT (Figure 1A). Viral replication assay results showed that CVV showed lower infection at 12 h, but showed higher replication rates subsequently, compared to EVV, which means vTK deficiency gives higher antitumor activity (Figure 1D). CVV and other engineered VV can be compared. Cytotoxicity results showed that CVV has more enhanced cytotoxicity in HepG2 cell than other VV deficient of vTk (e.g. JX-594), confirming that acquired cancer favoring capacity from evolution in tumor tissues gives more anti-tumor effects. Biodistribution results of CVV in tumor tissues vs normal tissues, higher replication rates in cancer cells than in normal cells and no cytotoxic effects on mES (mouse embryonic stem cells) in Supplementary Figure 1 confirmed that the higher cancer favoring affinity of CVV can provide much higher cancer selectivity.

### The CVV has higher cytotoxicity than conventional anticancer drugs on different types of HCC

**Figure 2:**
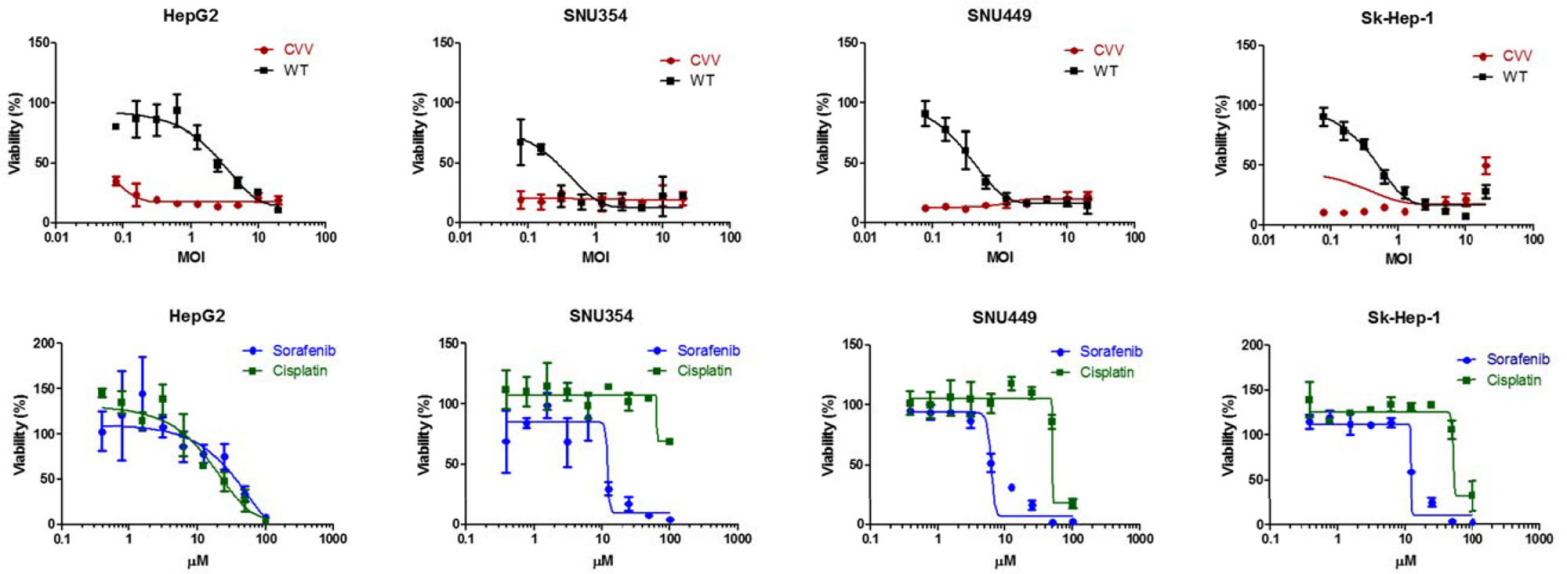
The cytotoxicity results of CVV and anticancer drugs on HCC CVV showed higher toxicity than WT. Vaccinia virus showed high toxicity in these cell lines even in a low dosage treatment; however, the general anticancer drugs sorafenib and cisplatin do not work very well up to ∼10 μM and100 μM respectively. CVV, cancer-favoring engineered vaccinia virus; MOI, multiplicity of infection; WT, wild type.

To demonstrate the cancer-favoring and oncolytic potency of CVV, a panel of HCC cell lines (i.e., HepG2, SNU354, SNU449 and Sk-Hep-1) was tested. When the four HCC cell lines were infected, the CVV showed higher toxicity than the WT (Figure 2, upper row). Although a direct comparison of the viral MOI and drug μM concentration is impossible, it is safe to assume that the compared virus concentration was much lower than drug concentration used when considering their approximate molar concentration (i. e. 1 MOI *vs* 10 μM). Vaccinia virus showed high toxicity in these cell lines even in a low dosage treatment; however, the general anticancer drugs sorafenib and cisplatin do not work very well up to ∼10 μM and 100 μM respectively (Figure 2, bottom row) whereas did work in HepG2 in a dose-dependent manner. The observation of elevated cell viability (over 100%) after the 72 h-CPT11 or sorafenib treatment may have been because WST-1 measure dehydrogenase activity, which is generally elevated in cancer stem cells.

### The CD44 and cellular thymidine kinase (cTk) expressions in HCC

**Figure 3:**
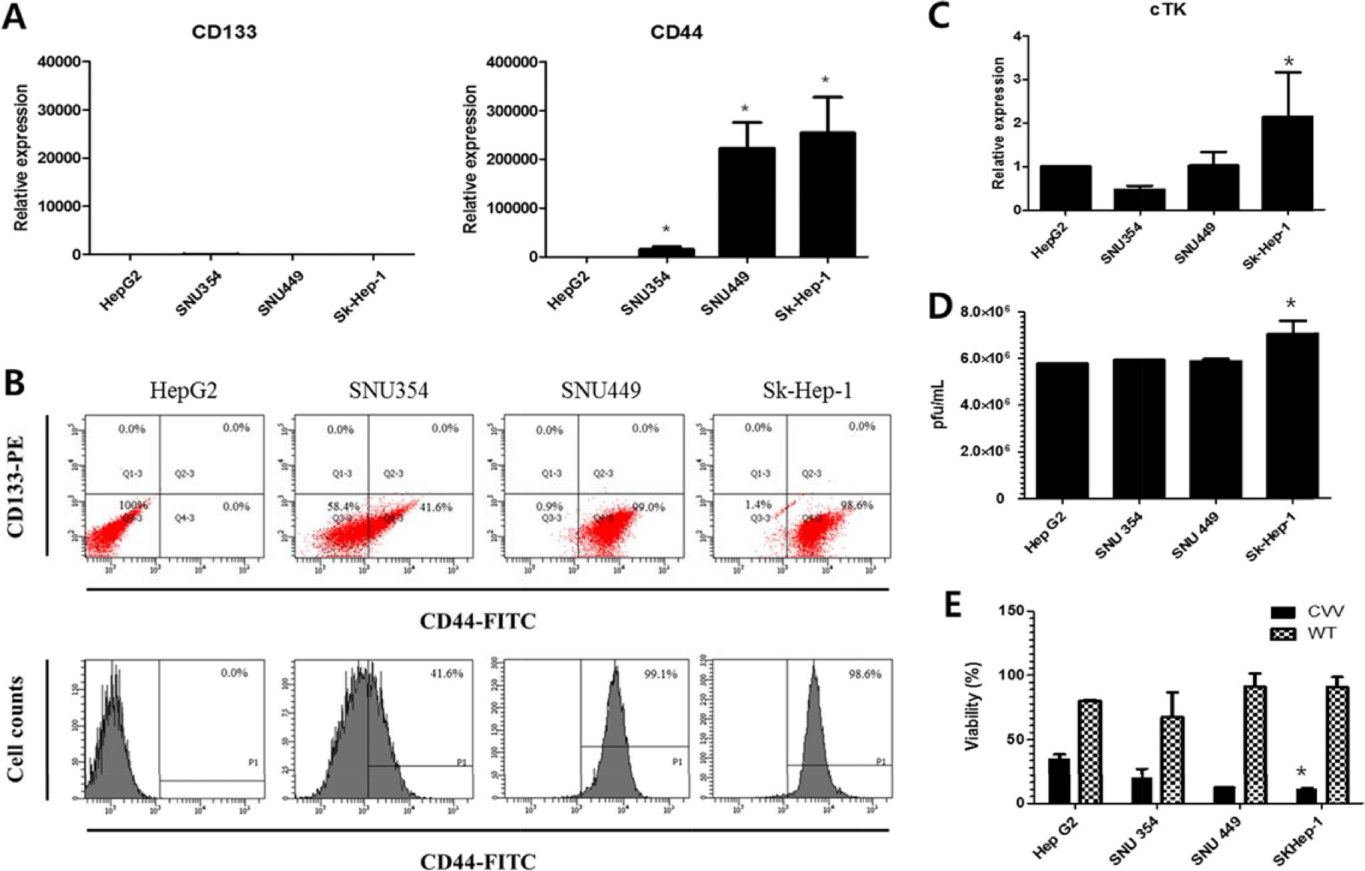
CD44 and cTk expression in different HCC cell lines (**A**) The real-time polymerase chain reaction results of the expression of CD133 and CD44. The CD44 expression level was least in HepG2, followed by (in increasing order) SNU354, SNU449, and Sk-Hep-1. (**B**) The flow cytometry results of the CD44 expression. The CD44 cell populations in HepG2, SNU354, SNU449 and Sk-Hep1 were 0%, 41.6%, 99.1% and 98.6%, respectively. (**C**) cellular thymidine kinase (cTk) expression, (**D**) replication efficiency and (**E**) cytotoxicity of CVV in HCC cell lines (*n = 3, *p* < 0.05). CVV affinity may be related with cTK expression of host cells because of vTk deficiency of CVV. Interestingly, cTk expression was also highest in Sk-Hep-1. Replication assay result shows that gradual increase of viral replications in HepG2, SNU354, SNU449, and also highest in Sk-Hep-1. And cytotoxicity of CVV was also highest in Sk-Hep-1.

We next examined the real-time PCR results of the expression of the cancer stem cell markers CD133 and CD44 in these cells (Figure 3A). The relative expression shows that the expression levels of CD44 were significantly higher than the expression level of CD133, which was again confirmed by FACS analysis (Figure 3B). The CD44 expression level was least in HepG2, followed by (in increasing order) SNU354, SNU449, and Sk-Hep-1. Relative RNA expression level of CD44 in SNU354, SNU449 and Sk-Hep-1 were ∼20,000,∼ 220,000 and ∼250,000, respectively (When the level of CD44 in HepG2 was set as 1). The CD44 cell populations in HepG2, SNU354, SNU449 and Sk-Hep1 were 0%, 41.6%, 99.1% and 98.6%, respectively. High resistance to anticancer drug in SNU354, SNU449 and Sk-Hep-1 seems related with the expression of CD44, a cancer stem cell marker in liver cells [15, 16] whereas responses to CVV in HCC does not. CVV affinity may be related with cTK expression of host cells because of vTk deficiency of CVV. Interestingly, cTk expression was also highest in Sk-Hep-1 (Figure 3C). Replication assay result shows that gradual increase of viral replications in HepG2, SNU354, SNU449, and also highest in Sk-Hep-1 (Figure 3D). And cytotoxicity of CVV was also highest in Sk-Hep-1 (Figure 3E).

### Cell migration and epithelial-mesenchymal transition (EMT) gene expression in HCCs

**Figure 4:**
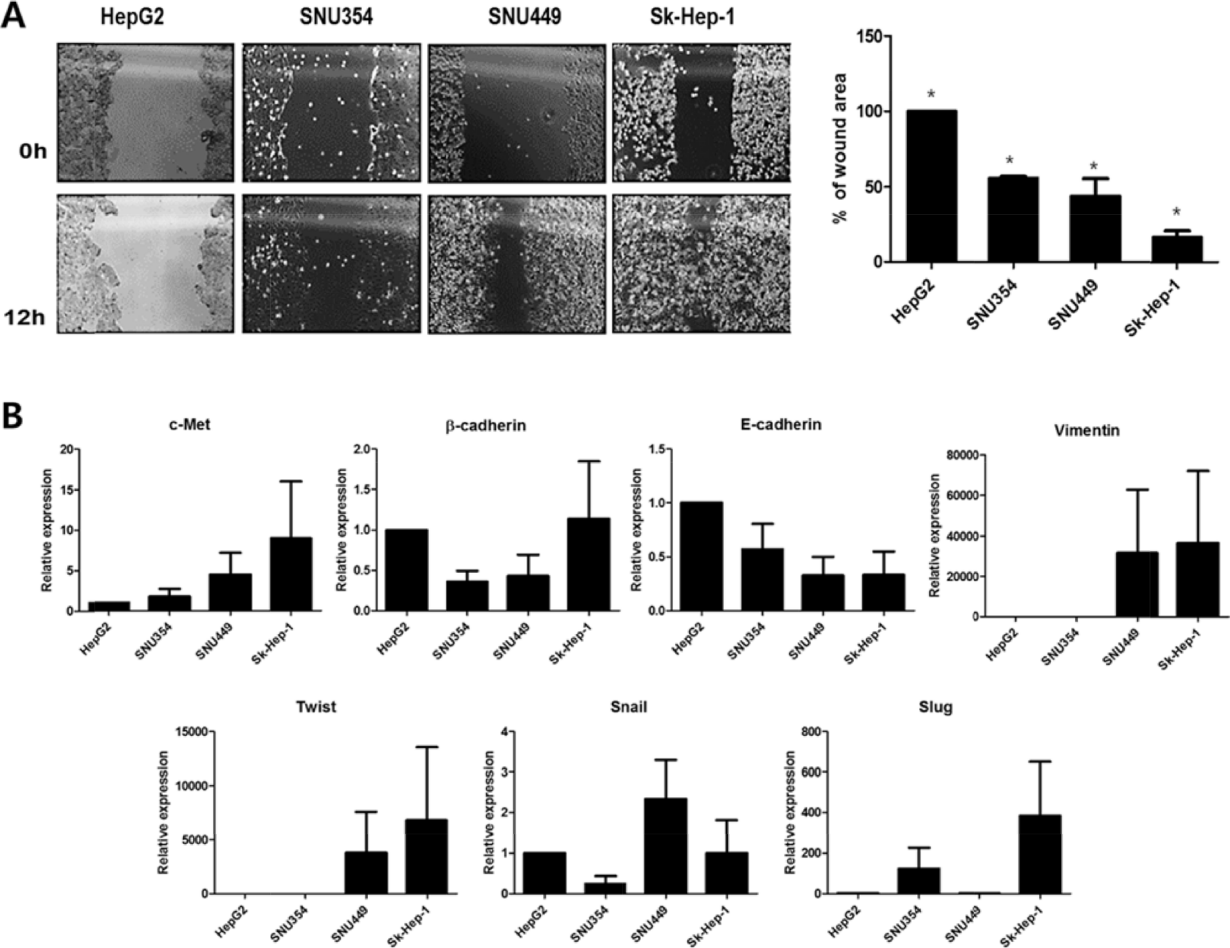
Cell migration and EMT gene expression of HCCs (**A**) The cell migration assay (*p < 0.05) and (**B**) EMT gene expression in four different HCCs. HCCs with higher CD44 expression shows higher migration rates and c-Met and EMT gene expression. EMT, epithelial-mesenchymal transition.

Cell migration and EMT gene expression in relation to previous finding of CD44 and cTk expression in HCC were then examined. To examine the relation of CD44 expression to liver metastasis, cell migration was determined by a wound-healing assay (Figure 4A). The migration capacity of the HCCs interestingly correlated with the CD44 expression level, which was least in HepG2, followed by (in increasing order) SNU354, SNU449, and Sk-Hep-1. A lower wound area percentage was associated with higher migration. This finding indicates that the highest CD44-expressing cell line, Sk-Hep-1, had the highest migration rate. Cell migration might reflect possibility of metastasis. Then, the expression of the metastatic marker c-Met and EMT genes such as β-cadherin, E-cadherin, Twist, Snail and Slug was examined (Figure 4B). Interestingly, the expression patterns of c-Met, which is associated with tumor invasion and metastasis [11, 12, 17] were similar to the previously found CD44 expression patterns in HCC. The correlation between CD44 expression and c-Met expression bring us that the migration activity of CD44 expressing stem cells would lead tumor invasion and metastasis [18]. Although no such a direct correlation between expression pattern in EMT expression and those of CD44 as found in 4 HCC cell lines, which is maybe because other upstream proteins such as Wnt, TGFβ etc are also involved in EMT expression [19], we found that CD44 expressing cells have generallly have high EMT expression which might be related to liver metastasis [20].

### The CD44 expression in relation to liver tumorigenicity and metastasis

**Figure 5:**
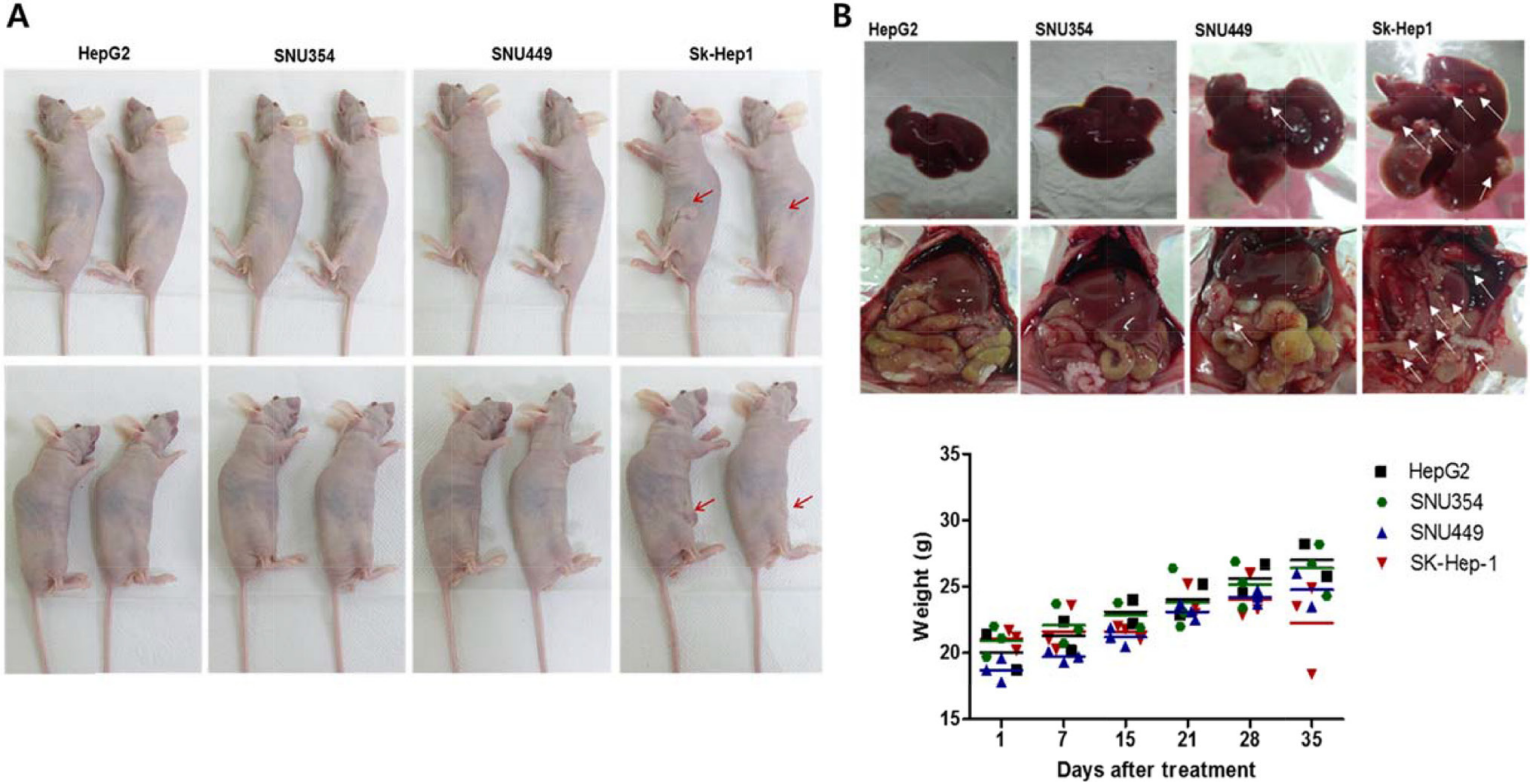
The CD44 expression in relation to liver tumorigenicity and metastasis (**A**) The tumorigenicity of Sk-Hep-1 cells. When the cells (2 × 10^6^ cells/100 μl) were subcutaneously injected into the mice, only Sk-Hep-1 induced tumor formation after 3 weeks. (*n* = 4) (**B**) Metastasis of directly injected HCCs to colon. When cells (2 × 10^6^ cells/100 μl) were directly injected into liver tissues, the hepatic tumor formation rate was also least in HepG2, followed by (in increasing order) SNU354, SNU449, and Sk-Hep-1. Hepatic metastasis to the colon region occurred only in the SNU449 and Sk-Hep-1 cell lines. Weight loss was also observed in SNU449 and Sk-Hep-1 injected mice (*n* = 4).

Liver tumorigenicity and meatastastic characteristics were also correlated with CD44 expression. When the cells (2 × 10^6^ cells/100 μl) were subcutaneously injected into the mice, only Sk-Hep-1 induced tumor formation after 3 weeks (Figure 5A, *n* = 4 in each group). In the previous studies using HepG2 for tumor formation [21], HepG2 cells at 10^6^ cells/mouse could not form visible xenografts in nude mice, but its sphere-forming cells at the same amount of sphere forming cells could form xenograft tumors and CD44 expression was found in HepG2 sphere forming cells whereas no CD44 expression in monolayer cultured HepG2 parent cells. It suggests that CD44 expression may account for cell tumorigeneicity. In this study, when cells (2 × 10^6^ cells/100 μl) were directly injected into liver tissues, the hepatic tumor formation rate was also least in HepG2, followed by (in increasing order) SNU354, SNU449, and Sk-Hep-1. Hepatic metastasis to the colon region occurred only in the SNU449 and Sk-Hep-1 cell lines (Figure 5B upper row). Weight loss was also observed in SNU449 and Sk-Hep-1 injected mice (Figure 5B, bottom row).

### The CD44 inhibition and CVV treatment attenuated cell migration and EMT expressions

**Figure 6:**
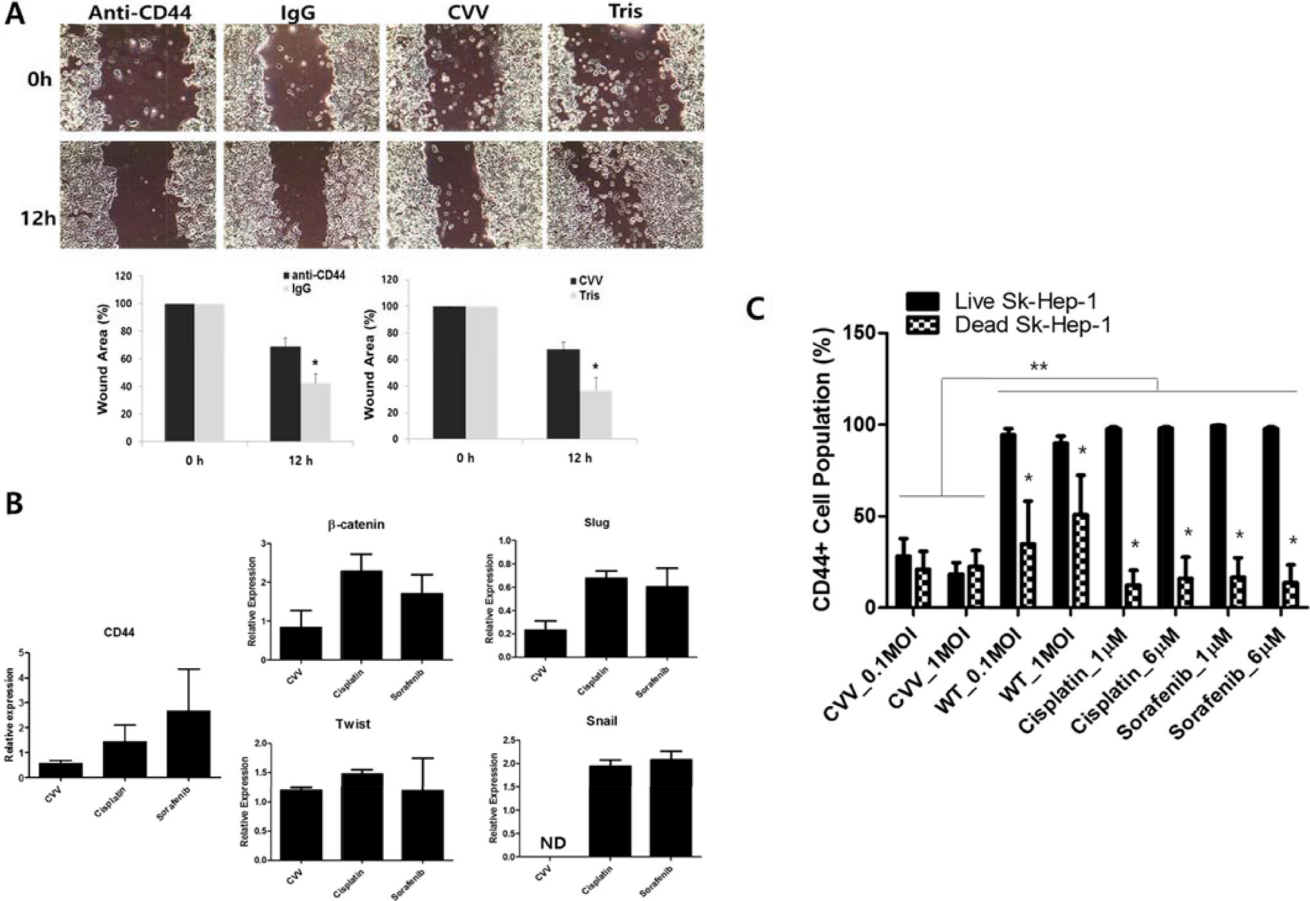
Inhibitory effects of anti-CD44 and CVV on cell migration (**A**) Sk-Hep-1 migration rate after anti-CD44 or CVV treatment. Cellular migration was inhibited when anti-CD44 or CVV was treated. IgG and Tris was treated as control. (*p < 0.05) (**B**) The expression of CD44 and the EMT markers in virus-treated and anticancer drug-treated Sk-Hep-1 cells. CVV treatment (1 multiplicity of infection, MOI) shows greater attenuation in EMT expression as well as in CD44 expression, compared to the target agent sorafenib (6 μM). (**C**) CD44+ cell population in live or dead Sk-Hep-1 cell after CVV, WT, cisplatin or sorafenib treatment. CD44+ population from either live cell fraction or dead cell fraction was decreased after CVV treatment whereas CD44+ populations only from dead cells was decreased. *p < 0.0001 (live *vs* dead), **p < 0.0001 CVV, cancer-favoring engineered vaccinia virus; IgG, immunoglobulin G.

The role of CD44 in cell migration was studied with anti-CD44-treated Sk-Hep-1 cells. Anti-CD44 treatment induced attenuated Sk-hep-1 migration (Figure 6A upper left). In addition, our CVV treatment showed attenuated migration (Figure 6A upper right). The control experiment for anti-CD44 and CVV treatment was done with anti-IgG and Tris buffer (because virus is constituted in pH 9.0 Tris buffer) treatment, respectively. Wound area measurement showed that anti-CD44 and CVV could attenuate cell migration (Figure 6A bottom row). The RNA expressions of β-catenin, slug, Twist and snail, which are well-known EMT markers, were examined after CVV, cisplatin, or sorafenib treatment. As Figure 6B demonstrates, CVV treatment (1 multiplicity of infection, MOI) shows greater attenuation in EMT expression as well as in CD44 expression, compared to the target agent sorafenib (6 μM) (Figure 6B). To confirm that the inhibitory migration ability of CVV on cell migration is not merely due to the cell death, we monitored the change in CD44 expressing pattern before and after CVV treatment and analyze the percentage of CD44 expressing cancer cell population in the remnant live cells after treatment (Figure 6C). Interestingly, CD44+ population from either live cell fraction or dead cell fraction was decreased after CVV treatment whereas CD44+ populations from dead cells after WT or anti-cancer drugs (cisplatin, sorafenib) treatment was only decreased, showing that CVV can successfully kill CD44+ cell populations, implying that the decreased CD44+ expression in live cells by CVV is the reason for the attenuated migration. High CD44+ cell population remaining in live Sk-Hep-1 after anti-cancer reagent may explain the resistant response of HCC to conventional cancer therapy, which are contributed by CD44 expression. Based on these results, CD44+ cells are associated with cellular migration, and thus metastasis, by affecting EMT expression, which can be further attenuated by CVV as well as anti-CD44. In term of cellular based assay, target agents such as sorafenib, or possibly anti-CD44 would give a good outcome, but there can be limitations when they are systemically treated. Here, CVV may target and kill metastatic cancers with its cancer-favoring characteristics.

### Cancer-favoring engineered vaccinia virus induced the complete regression of liver tumorigenicity and metastasis to the colon

**Figure 7:**
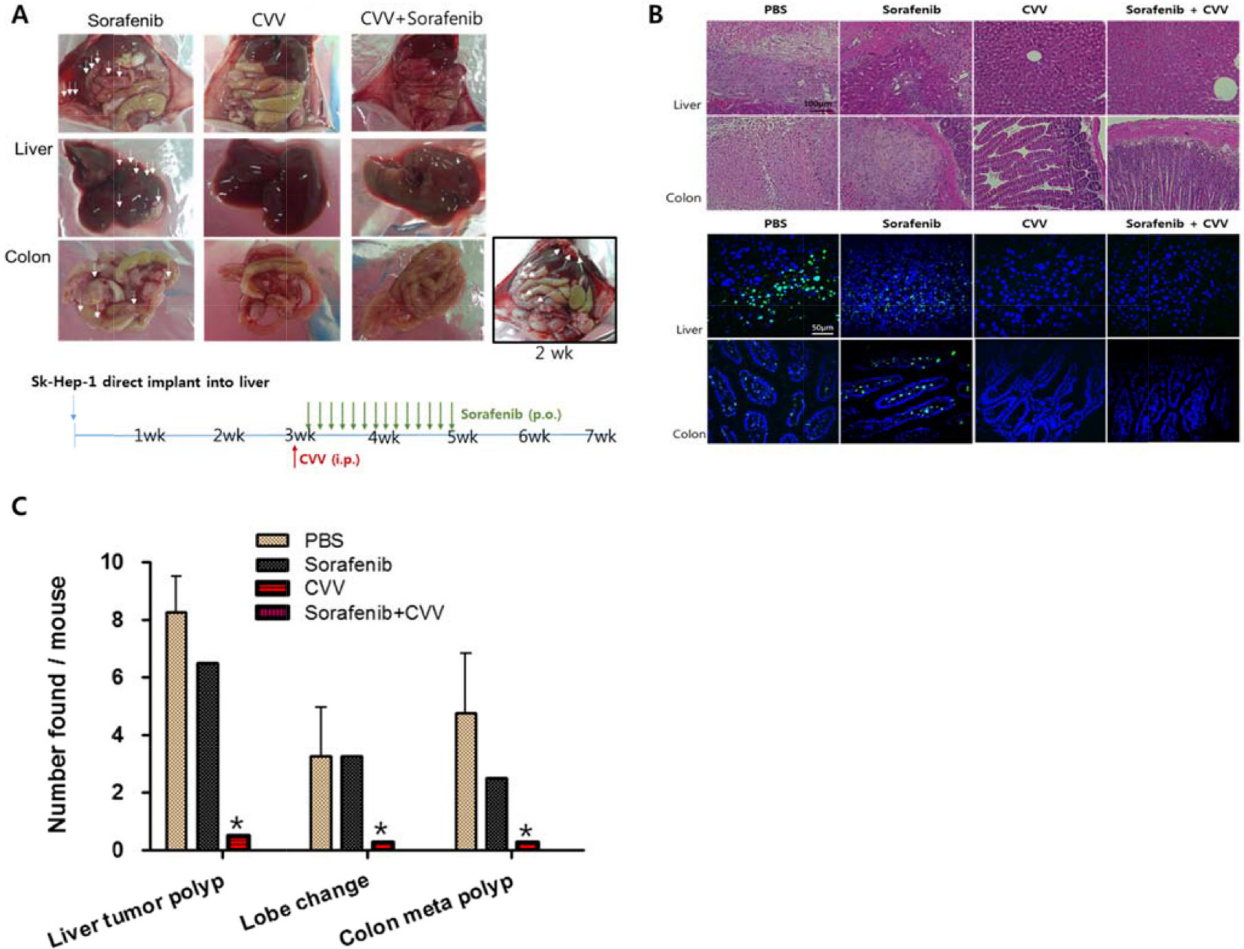
CVV induced complete regression of liver tumorigenicity and metastasis to colon (**A**) The metastasis of liver-injected Sk-Hep-1 cells to colon in Sorafenib, CVV or CVV+sorafenib treatment group. Four weeks after treatment (*n* = 4 in each group), metastatic regions were rare in the CVV-treated groups (i.e., CVV or sorafenib with CVV), whereas metastatic regions remained in the sorafenib-treated regions. (**B**) Hematoxylin and eosin staining and Tunnel assay results. (**C**) Number of liver tumor polyps, changed lobes, colorectal metastasized polyps observed in each mouse. (*n* = 4), *p < 0.0001. The complete regression of liver tumorigenicity and colorectal metastasis in the CVV-treated groups. CVV, cancer-favoring engineered vaccinia virus; PBS, phosphate-buffered saline; i.p., intraperitoneal; p.o., oral administration.

The effect of CVV or the anticancer drug sorafenib and cisplatin on liver tumorigenicity and colorectal metastasis was investigated *in vivo*. The highest metastatic and tumorigenic SK-Hep-1 cells were selected and injected directly into the liver tissue of Balb/c nude mice to induce liver-to-colon metastasis. After 2 weeks, liver tumorigenicity and metastasis to the colon were confirmed (Figure 7A right bottom corner). The mice were divided into four groups (i.e., PBS, sorafenib, CVV, or sorafenib with CVV). Four weeks after treatment, the metastatic regions in each group (*n* = 4) were examined. Sorafenib, CVV, and sorafenib with CVV treatment in the animal models were administered *via* the oral, peritoneal, or oral/peritoneal routes (Figure 7A bottom). As expected, metastatic regions, interestingly, were rare in the CVV-treated groups (i.e., CVV or sorafenib with CVV), whereas metastatic regions remained in the sorafenib-treated regions (Figure 7A, upper row). Hematoxylin and eosin (H&E) staining (Figure 7B, upper row) and terminal deoxynucleotidyl transferase dUTP nick end labeling (TUNEL) staining (Figure 7B bottom row) of the liver and colon tissue sections of each sample confirmed the complete regression of liver tumorigenicity and colorectal metastasis in the CVV-treated groups. Number of liver tumor polyps, changed lobes, colorectal metastasized polyps observed in each mouse was presented in Figure 7C.

## DISCUSSION

Molecular-targeted agents have recently been introduced into clinical use for treating HCCs [22, 23]. However, HCCs are generally refractory to many targeted therapies because of the many genetic alterations and abnormalities that contribute to tumor development and progression from tumor to tumor [24, 25]. Sorafenib is the only effective systemic treatment for advanced HCC, but it only prolongs survival duration for approximately 3 months. Different molecular alterations influence patient response, depending on the underlying risk factors and etiologies of HCCs. Therefore, it is a major challenge to identify the key molecules, receptors, or signaling pathways and to assess their relevance as potential targets [26]. Here, we propose a simple and strategic virus CVV for treating metastatic HCCs. We found that CD44 expression was associated with drug resistance, tumorigenicity and cellular metastasis and CVV can successfully eradicate metastatic liver cancer cells.

The vaccinia virus (VV) is a well-known oncolytic virus that possesses cancer selectivity and efficiency in killing cancers [27–29]. Oncolytic virus-based therapy has recently shown promising results in cancer selectivity and safety in clinical trials [30–32]. Our previous study of a multicenter, multinational, randomized, phase 2, stratified, parallel-group dose-clinical trial in patients with advanced HCC showed that overall survival was higher in patients with advanced HCC who received high-dose treatment (10^9^ pfu) than in patients who received low-dose treatment (10^8^ pfu), providing that the treatment with the JX-594 virus is safe and well-tolerated; there were no treatment-related deaths [6].

**Figure 8:**
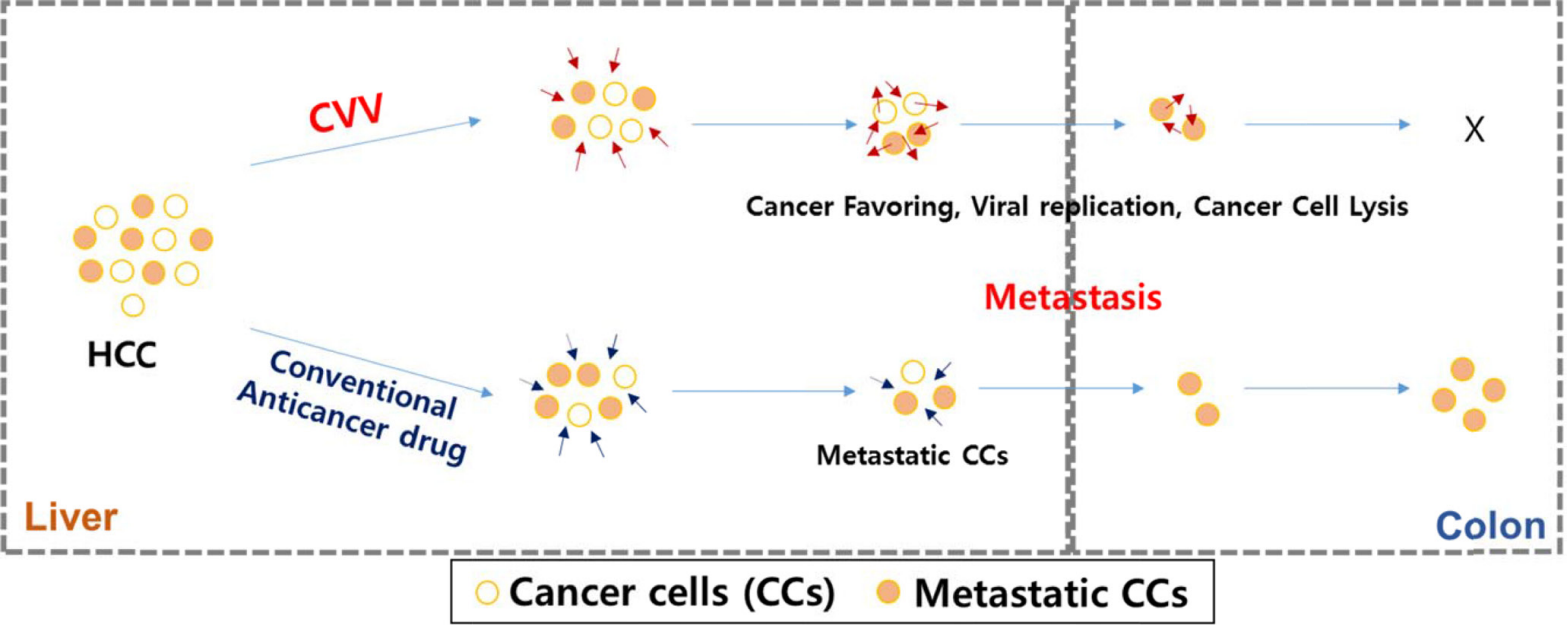
Schematic illustration of how CVV can target and kill the metastatic HCC by overcoming limited effects of anticancer drugs The CVV has enhanced cancer-favoring selectivity and cytotoxicity toward metastatic liver cancer stem cells with high EMT & cTk expression, because of its cancer-favoring characteristics and the different cytotoxic pathway used to kill cancer cells.

Based on the accumulated data on the safety and efficacy of previously reported oncolytic virus-based therapy and its cancer selectivity and the acquiring of higher cancer-favoring characteristics by the natural evolutional process and genetic deletion of vTK gene from the VV, we hypothesized that our developed CVV (Figures 1 and 2) could selectively track and eradicate metastasized cancer. This study attempted to examine the therapeutic efficacy of our CVV (which is a cancer-favoring oncolytic vaccinia virus produced by first evolving it from the tumor mass and then by inactivating the vTk gene) in attenuating and regressing metastatic HCCs. We developed a metastatic HCC animal model with the Sk-Hep-1 cell line, which highly expresses CD44 (Figures 3–5), and found that CVV can attenuate cell migration (Figure 6) and thus regress the metastasis of highly metastatic Sk-Hep-1 cells (Figure 7) by inducing lower CD44 and thus reduced EMT marker expression. We believe that the simple and strategic cancer-favoring characteristics of our CVV can effectively and selectively tract, infect, and replicate in metastatic HCCs and finally inactivate EMT expression and attenuate cell migration and metastasis because it favors cancer cells and is generally not affected by drug resistance pathways that are primarily attributed to cancer-initiating cells (Figure 8).

## MATERIALS AND METHODS

### Cell culture and reagents

The HCC cell lines HepG2, SNU354, SNU449, and Sk–Hep1 were obtained from the Korean Cell Line Bank (Seoul, South Korea). The cells were cultured in Roswell Park Memorial Institute (RPMI) 1640 medium, which was supplemented with 10% fetal bovine serum (FBS), and 100 U/mL penicillin and streptomycin under standard conditions of 37°C, 5% carbon dioxide, and humidified atmosphere. All culture media and supplements were obtained from Welgene (Daegu, South Korea). The anticancer drugs sorafenib and cisplatin were purchased from Selleckchem (Houston, TX, USA). Antibodies to all of the following proteins were used: CD44 (3578S; Cell Signaling, Beverly, MA, USA), CD44-FITC or APC (MACS, Surrey, UK), CD133-PE CD133 (BD, Piscataway, NJ).

### Cell proliferation (cytotoxicity) assay

Cells were seeded at 10,000 cells per well in 96-well plates. After 1 day, the cells were treated for 2 hours with the anticancer drug or oncolytic virus at the desired concentration in serum-free media. The media were then changed with normal culture media. At 24 hours, 48 hours, and 72 hours after treatment, cell viability was assessed by WST assay (EzCytotox; iTSBiO, Seoul, South Korea) in accordance with the manufacturer's instructions. The absorbance of each sample was measured using a microplate reader at 450 nm. The reference wavelength was 680 nm.

### Real-time polymerase chain reaction

Total ribonucleic acid (RNA) was extracted using TRIzol reagent (Life Technologies, Carlsbad, CA, USA) in accordance with the manufacturer's instructions. In brief, RNA purity was verified by measuring the 260/280 absorbance ratio. The first strand of complementary deoxyribonucleic acid DNA (cDNA) was synthesized with 2 μg of total RNA using the RH(−) RT Synthesis kit (iNtRON Biotechnology, Seongnam, South Korea). Two microliters of the cDNA was used for each polymerase chain reaction (PCR) mixture containing SYBR-Green qPCR mix (Roche, Basel, Switzerland). Real-time PCR was performed using the LightCycler 96 Real-Time PCR System (Roche). The reaction was subjected to 45-cycle amplification at 95°C for 10 seconds, at 60°C for 10 seconds, and at 72°C for 10 seconds. The relative mRNA expression of the selected gene was normalized to *beta*-actin and quantified using the ΔΔCt method. The primers used are listed in Table 1.

**Table 1:** Primers used in this study

Name	Sequences (5′ -> 3′)
CD44 Forward	AGAAGGTGTGGGCAGAAGAA
CD44 Reverse	AAATGCACCATTTCCTGAGA
CD133 Forward	GCATTGGCATCTTCTATGGTT
CD133 Reverse	CGCCTTGTCCTTGGTAGTGT
cMet Forward	GGTTGTGGTTTCTCGATCAGG
cMet Reverse	TTCGTGATCTTCTTCCCAGTGA
β-catenin Forward	AAAATGGCAGTGCGTTTAG
β-catenin Reverse	TTTGAAGGCAGTCTGTCGTA
Slug Forward	CTGGGCTGGCCAAACATAAG
Slug Reverse	CCTTGTCACAGTATTTACAGCTGAAG
Twist Forward	GCAGGACGTGTCCAGCTC
Twist Reverse	CTGGCTCTTCCTCGCTGTT
Snail Forward	TTCTCTAGGCCCTGGCTGC
Snail Reverse	TACTTCTTGACATCTGAGTGGGTCTG
β-Actin 184 Forward	AGAGCTACGAGCTGCCTGAC
β-Actin 184 Reverse	AGCACTGTGTTGGCGTACAG

### Flow cytometry

The HCC cell lines were washed in phosphate-buffered saline (PBS), which included 2% FBS. The suspension cells were incubated with fluorochrome-conjugated antibody at 4°C for 30 minutes. Flow cytometry (FACS) analysis was performed using the FACS Canto II system (Becton Dickinson, Franklin Lakes, NJ, USA). The FACS data were analyzed using FACS Diva software (Becton Dickinson (BD)). Antibodies to the following proteins were used: fluorescein isothiocyanate (FITC) or allophycocyanin (APC)-conjugated CD44 (MACS) and phosphatidylethanolamine (PE)-conjugated CD133 (BD). The FACS gates were established by staining with isotype antibody conjugated FITC, APC and/or PE (BD).

### Migration assay

Cells were seeded at 1 × 10^5^ cells per well onto 24-well plates. When the cells reached 100% confluence, a scratch was produced by a pipette tip. After being cultured for 12 hours, images were acquired in triplicate, and the data were measured as the average area of five random fields with Image J software (available at http://imagej.nih.gov/ij).

### *In vivo* HCC metastatic model

All mice were maintained in accordance with the Institutional Animal Care and Use Committee-approved protocols of the Pusan National University (Busan, South Korea; PNU-2014-0685). Nude Balb/c mice were purchased from Orient (Gpyeong, Korea). A HCC metastatic mouse model was formed using Sk-Hep1 cells. The cells (2 × 10^6^ cells/100 μL) were directly injected into the liver. After 2 weeks, the animals were divided into four groups and then treated by sorafenib (400 μg per mouse, daily for 2 weeks), CVV (10^6^ pfu per mouse, once weekly), sorafenib (400 μg per mouse, daily for 2 weeks) plus CVV (10^6^ pfu per mouse, once weekly), or PBS (daily for 2 weeks) as the control. The doses of sorafenib and CVV were chosen based on the previous study [9, 13]. After 1 month of treatment, the mice were sacrificed, and their tissues were immediately fixed in 4% paraformaldehyde.

## CONCLUSION

By taking these factors together, we concluded that our simple and cancer-favoring strategic virus design of the CVV eradicates metastatic CD44-expressing cells, provided that our CVV may be a promising therapeutic reagent that targets metastatic liver cancer cells.

## SUPPLEMENTARY FIGURE


