# Supplementary material for: Evolutionary cancer-favoring engineered vaccinia virus for metastatic hepatocellular carcinoma
Source: Oncotarget. 2017 Apr 20;8(42):71489–99. doi: 10.18632/oncotarget.17288 (PMC5641064; doi:10.18632/oncotarget.17288)
Supplement: Supplementary file 1 [file oncotarget-08-71489-s001.pdf]

Evolutionary cancer-favoring engineered vaccinia virus for metastatic hepatocellular carcinoma

Supplementary Materials

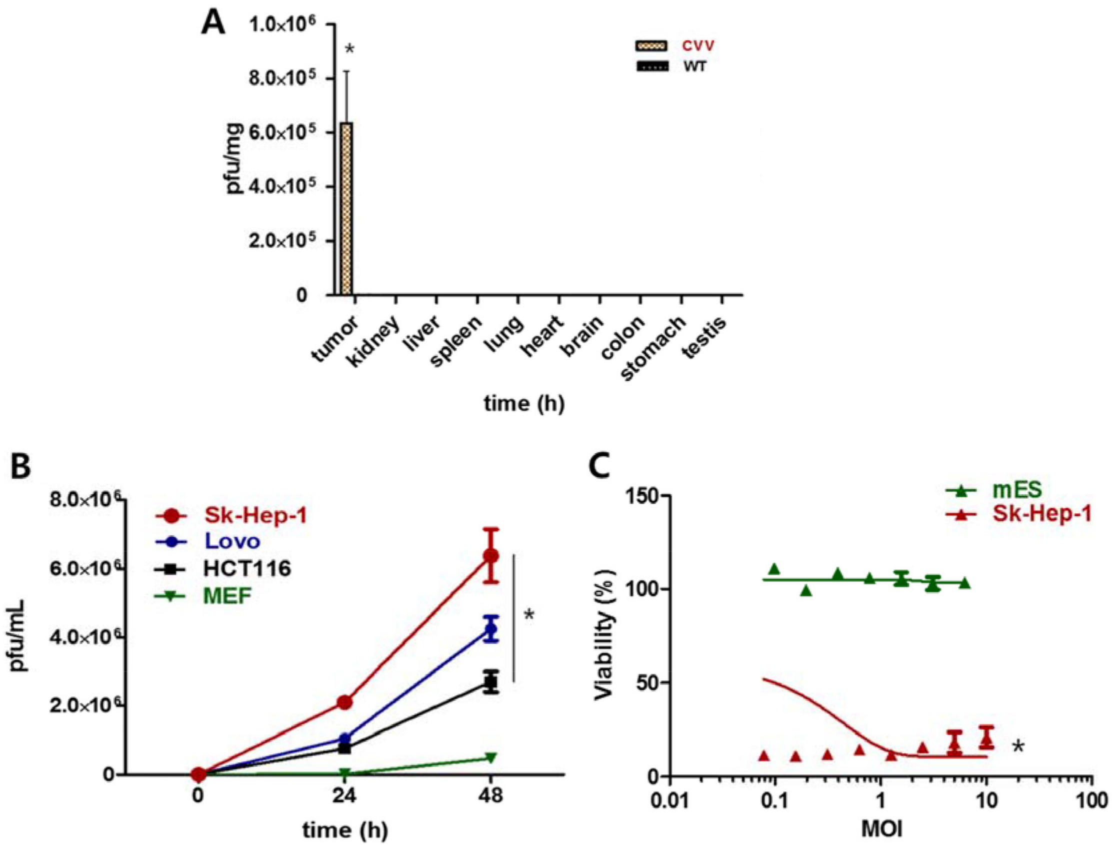

Supplementary Figure 1: Cancer favoring affinity of CVV. (**A**) Biodistribution results at 2 weeks post-intraperitoneal injection of virus in HT29-bearing mice, showing that CVV selectively infected tumor masses. (*$p < 0.0001$). (**B**) Higher replication efficacy in cancer cells (Sk-Hep-1: human hepatocellular carcinoma cells, Lovo & HCT116: human colon cancer cells) than in normal cells (MEF: mouse embryonic fibroblast cells) (*$p < 0.0001$ vs MEF). (**C**) Cytotoxicity results of CVV on normal mouse embryonic stem cells (mES) vs human hepatocellular carcinoma, Sk-Hep-1 (*$p < 0.0001$ vs mES).
